# Haemoperitoneurn Secondary to Rupture of Retroperitoneal Variceal

**DOI:** 10.1155/1997/65261

**Published:** 1997

**Authors:** M. Molina-Perez, F. Rodriguez-Moreno, E. Gonzalez-Reimers, J. Perez-Palma, F. Santolaria-Fernandez, H. Essardas-Daryanani, A. Martinez-Riera

**Affiliations:** 1 Dpto. de Medicina Interna y Cirugía General Tenerife Canary Islands La Laguna Spain; 2 Hospital Universitario de Canarias Tenerife Canary Islands La Laguna Spain

## Abstract

A 45-year-old alcoholic male patient presented with hypovolemic shock and intense anemia (Hemoglobin 04.7 g/dl), and was operated on. A bleeding retroperitoneal varix located near the right colon was responsible for the clinical picture and was sutured. After operation the patient developed haemodynamic instability and pneumonia a situation which was reverted with intensive medical therapy. The patient is now doing well.


*HPB Surgery*, 1997, Vol. 10, pp. 329–331
Reprints available directly from the publisher
Photocopying permitted by license only

© 1997 OPA (Overseas Publishers Association)
Amsterdam B. V. Published in The Netherlands
by Harwood Academic Publishers
Printed in India

*Case Report*

### Haemoperitoneum Secondary to Rupture of Retroperitoneal Variceal

M. MOLINA-PEREZ[b], F. RODRIGUEZ-MORENO[b], E. GONZALEZ-REIMERS[b,*], J. PEREZ-PALMA[a], F. SANTOLARIA-FERNANDEZ[b], H. ESSARDAS-DARYANANI[b] and A. MARTINEZ-RIERA[b]

[a]*Dpto. de Medicina Interna y Cirugía General;* [b]*Hospital Universitario de Canarias, La Laguna, Tenerife, Canary Islands (Spain)*

*(Received 16 February 1996)*

**A 45-year-old alcoholic male patient presented with hypovolemic shock and intense anemia (Hemoglobin 04.7 g/dl), and was operated on. A bleeding retroperitoneal varix located near the right colon was responsible for the clinical picture and was sutured. After operation the patient developed haemodynamic instability and pneumonia a situation which was reverted with intensive medical therapy. The patient is now doing well.**

*Keywords:* Haemoperitoneum, variceal bleeding, portal hypertension

Variceal bleeding is a common complication of portal hypertension. These varices are usually located in the esophageal submucosa, although bleeding from ectopic varices has also been described [1–3]. We present here the case of a patient in whom hemoperitoneum and hypovolemic shock followed bleeding from retroperitoneal varices.

### CASE REPORT

A 45 year-old, heavily alcoholic, male patient was admitted to our Hospital because of jaundice for several days nausea and vomiting dark material on the day of admission. Physical examination was consistent with ascites, jaundice, pallor and tachycardia (120 beats/minute). Laboratory evaluation showed intense anemia (hemoglobin = 4.7 g/dl, red blood cell count = $1{,}31 \times 10^{12}/1$) and low prothrombin activity (31%). During evaluation in the emergency room, the patient become profoundly hypovolemic, with a collapsable pulse, tachycardia and profuse sweating, followed by respiratory arrest. After resuscitation, bloody ascitic fluid was obtained by paracentesis, and the patient was rushed to the operating room.

When the abdominal cavity was entered, 4 liters of haemorrhagic fluid were encountered. After drainage, a cirrhotic liver was observed, together with grossly dilated retroperitoneal varices, one of which was oozing blood. These

*Author for correspondence.

varices were located in the right lateral wall of the abdominal cavity, near the ascending colon. After suture of the bleeding varix, the abdomen was closed. Postoperatively, the patient progressively developed haemodynamic instability, tachypnea and hypoxemia despite progressive increase in inspiratory oxygen fraction, a "white lung" being observed on the chest X-ray. However, with adequate fluid and blood replacement, dopamin and positive end-expiratory pressure, the patients general status improved, extubation being possible 25 days later. The patient was discharged a few days later, and is doing well–except for the fact that he is drinking again.

### DISCUSSION

Portal hypertension results in the development of porto-systemic collaterals. Portosystemic communications exist as a rich network of very fine vessels which become dilated when portal hypertension develops [4]. Usually, these collaterals are seen at the gastroesophageal union, anorectal plexus, and umbilical vein, leading to oesophageal varices and haemorroids, and to the classic collateral circulation in the abdomen, although retroperitoneal veins (Retzius veins) connecting colic veins with lumbar and lower intercostal ones, and Sappey veins, consisting of numerous channels between liver and diaphragm, are also well known. However, varices also develop where organs supplied by the splachnic circulation contact the retroperitoneum [4], and also in organs with tributaries to the inferior and superior mesenteric veins [3]. Bleeding from varices located in these places result in colonic, duodenal, small intestinal and even vaginal bleeding [5–9]. Rupture of extra-intestinal varices, around ascending and descending colon, duodenum and pancreatic region, posterior aspect of the liver, and posterior to the spleen- is a rare, lifethreatening complication associated with high mortality rates [10]. Indeed, until 1982, only 14 cases of haemoperitoneum secondary to variceal bleeding had been described, with a mortality rate of 78%, partly due to the fact that bleeding at these sites usually results in profound hypovolemic shock [1].

The main cause of haemoperitoneum in liver cirrhosis is liver rupture due to hepatocellular-carcinoma, this complication being usually the final event of this disease [11]. However, it is important to keep in mind that the possibility of oozing ectopic varices exist, and that prompt surgery and intensive medical support may result in survival of this otherwise ominous complication.

### *References*

[1] Fox, L., Crane, S. A., Bidari, C. and Jones, A. (1982). Intraabdominal hemorrhage from ruptured varices. *Arch. Surg.*, **117**, 953–956.

[2] Gudjonsson, H., Zeiler, D., Gamelli, R. L. and Kaye, M. D. (1986). Colonic varices. Report of an unusual case diagnosed by radionuclide scanning, with review of the literature. *Gastroenterology*, **91**, 1543–1547.

[3] Hamlyn, N. A., Lunzer, M. R., Morris, J. S., Puritz, H. and Dick, R. (1974). Portal hypertension with varices in unusual sites. Lancet (II), 1531–1534.

[4] Edwards, E. A. (1951). Functional anatomy of the portal-systemic communications. *Arch Intern Med.*, **88**, 137–154.

[5] Lopata, H. E. and Berlin, L. (1966). Colon varices: a rare cause of lower gastrintestinla bleeding. *Radiology*, **87**, 1048–1050.

[6] Hermann, R. E. and Esselstyn, C. B. Jr (1967). The potential hazard of pregnancy in extrahepatic portal hypertension: report of two cases. *Arch. Surg.*, **95**, 956–959.

[7] Doberneck, R. C. and Janovski, N. A. (1970). Isolated bleeding from colonic varices in patients with liver disease. *Am. J. Dig. dis.*, **15**, 834–841.

[8] Kreek, M. J., Raziano, J. V. and Hardy, R. E. *et al.* (1967). Portal hypertension with bleeding vaginal varices. *Ann. Intern. Med.*, **66**, 756–759.

[9] Feu, F., Salmerón, J. M., Bruix, J., Gin'es, A., García-Valdecasas, J. C., Terés, J. and Rodés, J. (1988). Hemorragia por varices ectópicas por hipertensión portal. *Gastroenterología y Hepatología*, 426–428.

[10] Shapero, T. F., Bourne, R. H. and Goodall, R. G. (1978). Intraabdominal bleeding from variceal vessels in cirrhosis. *Gastroenterology*, **74**, 128–129.

### COMMENTARY

This is a case report of a middle-aged alcoholic man presenting with hypovolemic shock. There were no signs of gastrointestinal bleeding, but despite this the patient apparently was in hemorrhagic shock. The suspicion of intraabdominal hemorrhage was clarified upon paracentesis when bloody ascitic fluid was obtained. The patient was rushed to the operating theatre, and at operation bleeding from a dilated retroperitoneal varix was found. This was easily taken care of. Postoperatively the patient developed ARDS and eventually recovered.

It is important to recognize the possibility of intraabdominal extraintestinal varices in this group of patients. Although portal systemic collaterals are most frequently developed within the GI tract the authors clearly point out the possibility of extraintestinal varices. The presence of these collaterals rarely solves? causes? any problems but may eventually rupture, e.g., as a consequence of a mild trauma and since the bleeding is easily controllable one should not hesitate to perform an early laparotomy.

Prof. B. W. Jeppsson

Department of Surgery

Malmö University Hospital

Malmö

S-205 02

SWEDEN

